# Adult-Onset Acute Disseminated Encephalomyelitis with Epstein-Barr Virus Infection

**DOI:** 10.1155/2022/6149501

**Published:** 2022-06-04

**Authors:** Emika Murasawa, Masazumi Matsuda, Koichi Ishiyama, Tetsugaku Shinozaki, Toshiki Murata, Manabu Hashimoto

**Affiliations:** Department of Radiology, Akita University Graduate School of Medicine, Akita, Japan

## Abstract

We present the case of a 22-year-old man who was diagnosed with tonsillitis and treated with antibiotics. Although the symptoms subsided, 1 week later, he presented with weakness in the lower limbs and was hospitalized. The weakness in the lower limbs worsened; he developed difficulty speaking and was transferred to our hospital. Laboratory tests showed a white blood cell count of 10,600/*μ*L (24% atypical lymphocytes). Positive results were obtained for immunoglobulin M (IgM) antibody against Epstein-Barr virus (EBV) viral capsid antigen. EBV-deoxyribonucleic acid quantification in blood yielded positive results. Magnetic resonance imaging (MRI) revealed a hyperintensity in the spinal cord at the Th11 level of the lower spine on T2-weighted imaging (T2WI). In addition, T2WI and fluid-attenuated inversion recovery imaging showed hyperintense lesions on the right cerebral peduncle, bilateral thalami, posterior leg of the left internal capsule, and right corona radiata. We diagnosed acute disseminated encephalomyelitis (ADEM) with EBV and initiated steroid pulse therapy. Symptoms, along with the lesions seen on MRI, subsequently ameliorated. This case suggests that ADEM can be difficult to diagnose, but careful diagnosis is crucial since appropriate treatment is necessary to improve the symptoms.

## 1. Introduction

Epstein-Barr virus (EBV) is a deoxyribonucleic acid (DNA) virus in the herpesvirus genus. This virus may be associated with the onset of infectious mononucleosis and lymphoma [1, 2]. EBV is known to cause neurological complications such as meningitis, encephalitis, cranial nerve palsy, myelitis, peripheral neuropathy, and Guillain-Barré syndrome [1, 2].

Acute disseminated encephalomyelitis (ADEM) is an acute demyelinating disease resulting from inflammation and autoimmune targeting of the central nervous system. This disease is more common in children than in adults [3], with an estimated annual incidence of 0.07–0.8 per 100,000 per capita in children [4]. However, the incidence of ADEM in adults remains unclear [3].

We report herein an adult case of ADEM triggered by EBV infection.

## 2. Case Presentation

A 22-year-old man with no relevant medical history developed a sore throat and fever. He was diagnosed with tonsillitis and was started on treatment with antibiotics. Thereafter, the symptoms subsided, but he experienced weakness in the lower limbs 1 week later. He, therefore, visited another hospital and was admitted. He was later transferred to our hospital after exacerbation of the weakness in the lower limbs and development of difficulty speaking.

The patient was afebrile, with a blood pressure of 134/76 mmHg and a heart rate of 75 beats/min. Neurological examination revealed difficulty speaking and weakness of the iliopsoas muscle. No involvement of the cranial nerve system was identified, and reflexes remained intact. No ataxia was evident.

Laboratory tests (Table 1) showed a white blood cell count of 10,600/*μ*L (53% neutrophils, 22% lymphocytes, and 24% atypical lymphocytes) and a C-reactive protein level of 1.39 mg/dl; no abnormalities of liver or kidney function were identified at the time of admission. In the hospital, alanine aminotransferase (ALT) slightly increased to 67 IU/ml. Because of the presence of atypical lymphocytes, the initial acute tonsillitis and fever were diagnosed as symptoms consistent with infectious mononucleosis [5].

Both IgM and immunoglobulin G (IgG) antibodies against EBV-viral capsid antigen (VCA) showed positive results. Levels of EBV-nuclear antibody (EBNA) were below the level of sensitivity, as were levels of immunoglobulin G for EBV-early antigen-diffuse-type and restricted-type antibodies. No elevation was found by comparing paired sera for IgG antibody against EBV-VCA during the onset and recovery phases. However, there was positivity for IgM antibody against EBV-VCA and EBNA negativity; thus, the diagnosis was EBV infection [6].

In peripheral blood, EBV was confirmed using real-time polymerase chain reaction (PCR). There were 2.1 × 10^1^ copies/10^6^ white blood cells, and this result was positive.

At the time of admission, cerebrospinal fluid revealed the following: white blood cell count, 46/mm^3^ (97% monocytes and 3% polymorphic neutrophils); protein, 71 mg/dl; glucose, 70 mg/dl; and negative results for EBV-DNA. Ten days after admission, cerebrospinal fluid analysis revealed the following: white blood cell count, 21/mm^3^ (100% monocytes); protein, 44 mg/dl; and glucose, 57 mg/dl.

**Figure 1:**
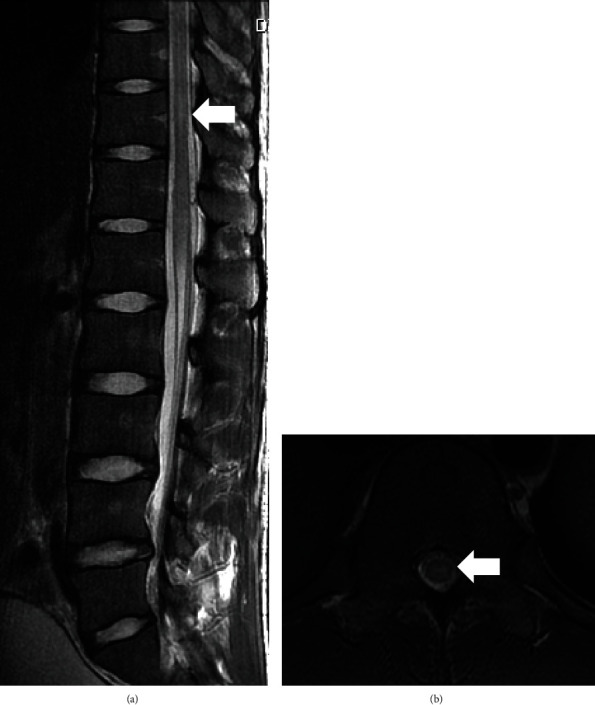
(a) Sagittal MRI T2-weighted imaging (T2WI) of the spine at the time of admission showed a hyperintense lesion in the spinal cord at the Th11 level of the lower spine (arrow). (b) Axial MRI T2-weighted imaging (T2WI) of the Th11 level of the spine at the time of admission showed a hyperintense lesion in the spinal cord (arrow).

**Figure 2:**
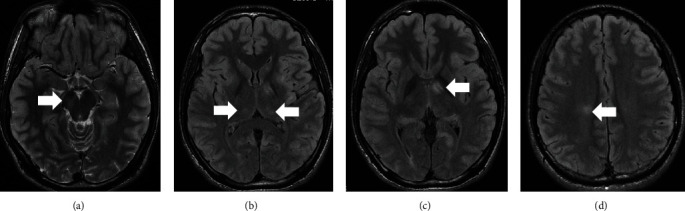
(a) T2WI imaging of the brain showed a hyperintense lesion at the right cerebral peduncle (arrow). (b) FLAIR imaging of the brain showed a hyperintense lesion at the bilateral thalami (arrow). (c) FLAIR imaging of the brain showed a hyperintense lesion at the left internal capsule (arrow). (d) FLAIR imaging of the brain showed a hyperintense lesion at the right corona radiata (arrow).

At the time of admission, magnetic resonance imaging (MRI) of the spine revealed a hyperintense lesion at the Th11 level of the lower spine on T2-weighted imaging (T2WI). No contrast effect was seen. MRI of the brain at the time of admission showed hyperintensities on the right cerebral peduncle, bilateral thalami, posterior leg of the left internal capsule, and right corona radiata on T2WI and fluid-attenuated inversion recovery (FLAIR) imaging (Figures 1 and 2). No contrast effect was seen.

No obvious abnormalities were evident from electroencephalography performed on day 5 of hospitalization.

Hepatosplenomegaly was observed on abdominal ultrasonography, with diameters of 15 cm for the liver and 11 cm for the spleen.

### 2.1. Progress after Hospitalization

We diagnosed infectious mononucleosis because he had acute tonsillitis, fever, and the presence of atypical lymphocytes. Furthermore, we diagnosed infectious mononucleosis due to EBV primary infection since the results showed positivity for IgM antibody against EBV-VCA and negativity for EBNA, although no elevation was found by comparing paired sera for IgG antibody against EBV-VCA during the onset and recovery phases.

Furthermore, we suspected ADEM-associated EBV based on the course of onset, the appearance of atypical lymphocytes, and results from MRI of the lower spine and brain and initiated steroid pulse therapy (methylprednisolone at 1,000 mg/day) for 5 days.

The patient showed improvements in speech from the day after starting steroid pulse therapy. After steroid pulse therapy had been performed for 3 days, other symptoms showed improvement. Communication with the patient normalized and steroid therapy was gradually reduced.

**Figure 3:**
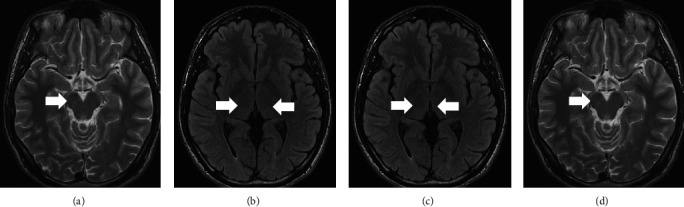
(a) T2WI imaging of the brain 12 days after admission showing the disappearance of the initial lesion (arrow). (b) FLAIR imaging of the brain 12 days after admission showing the disappearance of the initial lesion (arrow). (c) FLAIR imaging of the brain 12 days after admission showing the disappearance of the initial lesion (arrow). (d) FLAIR imaging of the brain 12 days after admission showing the disappearance of the initial lesion (arrow).

A follow-up brain MRI 12 days after admission showed the disappearance of the initial lesions (Figure 3). We diagnosed ADEM because of the amelioration of symptoms and lesion reduction seen on MRI achieved with steroid pulse therapy. The patient was discharged on hospital day 15.

## 3. Discussion

EBV is known to cause neurological complications, including meningitis, encephalitis, cranial nerve palsy, myelitis, peripheral neuropathy, and Guillain-Barré syndrome [1, 2]. Central nervous system complications of EBV infection occur in up to 18% of cases [7]. The frequency of ADEM is 0.4 per 100,000 capita for children, but the frequency for adults is unknown [3].

MRI has been reported as a useful diagnostic method. This modality shows multiple, disseminated lesions and white matter lesions [8, 9].

Various diagnostic criteria for ADEM have been proposed [10–13]. In these criteria, common findings for ADEM include acute-onset inflammatory lesions, multiple scattered lesions identified from clinical symptoms or MRI, monophasic lesions, and nonlocalized lesions such as optic neuritis or myelitis. However, no consensus has been reached on the presence or absence of prior infection, vaccination, cerebrospinal fluid findings, or MRI findings.

Direct infiltration of the virus into nervous tissue [7] or secondary immunological mechanisms triggered by viral infection could be considered the pathological conditions underlying ADEM [2]. In this case, because positive results were obtained for serum EBV-DNA, cerebrospinal fluid EBV-DNA was negative, and steroid pulse therapy proved effective; we attributed this case to an immunological mechanism secondary to direct infiltration of the virus into nerve tissues.

The treatment of ADEM is empirical, and high-dose corticosteroid (1 g/day) for 3–5 days has been reported to be useful [3, 11]. If steroids prove ineffective, intravenous immunoglobulins and plasma exchange are considered. More than 50% of patients show a good prognosis with appropriate treatment. If patients show convulsions or impaired consciousness on admission, the mortality rate can reach 25% [3]. ADEM thus requires prompt treatment.

In conclusion, we encountered a case of adult-onset ADEM with EBV. This pathology is rare and difficult to diagnose, but careful diagnosis is crucial since appropriate treatment is necessary to improve symptoms,

## Figures and Tables

**Table 1:** The patient's laboratory test results.

	Day of admission	14 days after admission	Reference range
*Hematology*			
White blood cells (/*μ*L)	10600	10200	4000-9000
Lymphocytes (/*μ*L)	2330	1820	
Lymphocyte (%)	22	17.8	26.6-46.6
Atypical lymphocytes (/*μ*L)	2540	0	
Atypical lymphocyte (%)	24	0	0-3.0
*Biochemistry*			
AST (IU/L)	26	24	13-33
ALT (IU/L)	41	67	8.0-42
*γ*-GTP (IU/L)	29	22	11-47
Total bilirubin (mg/dL)	0.4	0.6	0.2-1.2
Albumin (g/dL)	3.8	3.8	4.0-5.0
BUN (mg/dL)	13.1	15.3	8.0-22
Creatinine (mg/dL)	0.58	0.63	0.6-1.1
CRP (mg/dL)	1.39	0.02	0-0.19
*Immunoserology*			
Anti-EBV VCA IgG (times)	160	80	<10
Anti-EBV VCA IgM (times)	20	20	<10
EBNA (times)	<10	<10	<10
Anti-EA-DR IgG (times)	<10	<10	<10

Abbreviations: *γ*-GTP: gamma glutamyl transpeptidase; ALT: alanine transaminase; AST: aspartate transaminase; BUN: blood urea nitrogen; CRP: C-reactive protein; EA-DR: diffuse and restricted early antigen; EBNA: Epstein-Barr virus nuclear antigen; EBV: Epstein-Barr virus; IgG: immunoglobulin G; IgM: immunoglobulin M; VCA: viral capsid antigen.

## Data Availability

Our submission is a case report. So, the datas used to support the findings of this case report are included within the article. No extra data was used to support this study.

## Abbreviations

EBV: Epstein-Barr virus
DNA: Deoxyribonucleic acid
ADEM: Acute disseminated encephalomyelitis
MRI: Magnetic resonance imaging
T2WI: T2-weighted imaging
FLAIR: Fluid-attenuated inversion recovery.
